# Ambient Sensors for Elderly Care and Independent Living: A Survey

**DOI:** 10.3390/s18072027

**Published:** 2018-06-25

**Authors:** Md. Zia Uddin, Weria Khaksar, Jim Torresen

**Affiliations:** Department of Informatics, University of Oslo, 0316 Oslo, Norway; weriak@ifi.uio.no (W.K.); jimtoer@ifi.uio.no (J.T.)

**Keywords:** survey, ambient sensors, elderly

## Abstract

Elderly care at home is a matter of great concern if the elderly live alone, since unforeseen circumstances might occur that affect their well-being. Technologies that assist the elderly in independent living are essential for enhancing care in a cost-effective and reliable manner. Elderly care applications often demand real-time observation of the environment and the resident’s activities using an event-driven system. As an emerging area of research and development, it is necessary to explore the approaches of the elderly care system in the literature to identify current practices for future research directions. Therefore, this work is aimed at a comprehensive survey of non-wearable (i.e., ambient) sensors for various elderly care systems. This research work is an effort to obtain insight into different types of ambient-sensor-based elderly monitoring technologies in the home. With the aim of adopting these technologies, research works, and their outcomes are reported. Publications have been included in this survey if they reported mostly ambient sensor-based monitoring technologies that detect elderly events (e.g., activities of daily living and falls) with the aim of facilitating independent living. Mostly, different types of non-contact sensor technologies were identified, such as motion, pressure, video, object contact, and sound sensors. Besides, multicomponent technologies (i.e., combinations of ambient sensors with wearable sensors) and smart technologies were identified. In addition to room-mounted ambient sensors, sensors in robot-based elderly care works are also reported. Research that is related to the use of elderly behavior monitoring technologies is widespread, but it is still in its infancy and consists mostly of limited-scale studies. Elderly behavior monitoring technology is a promising field, especially for long-term elderly care. However, monitoring technologies should be taken to the next level with more detailed studies that evaluate and demonstrate their potential to contribute to prolonging the independent living of elderly people.

## 1. Introduction

**Figure 1:**
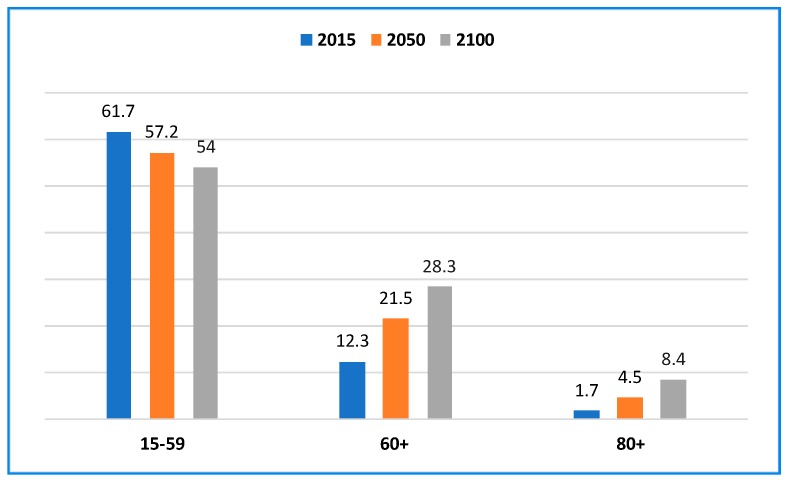
Percentages of persons of different ages in the world in different years [1].

Worldwide, the total number of elderly people is growing more rapidly compared to other age groups [1]. Consequently, the share of older persons is increasing almost everywhere. In 2015, one out of eight people worldwide was aged 60 years or over. By 2030, one out of six people will be in this age group globally. Furthermore, elderly people will outnumber children aged 0–9 years by 2030. By 2050, they may outnumber adolescents and youths aged 10–24 years. The aging process is more advanced in high-income countries. Japan has the most-aged population by far. In 2015, 33% of the population was aged 60 years or over. Regarding elderly population, Japan is followed closely by Germany (28%), Italy (28%), and Finland (27%). Therefore, the pace at which the world population is aging is increasing over time. By 2030, older persons are anticipated to account for substantially more than 25% of the populations in Europe and Northern America, 20% in Oceania, 17% in Asia, and 6% in Africa. As shown in Figure 1, the elderly population (aged 60 years or more) will increase faster as a percentage of the total population than the population of ages 15–59 years. If the trend continues, there will not be enough people to take care of the elderly in the distant future. Hence, assisted living technologies will be needed in the future to take care of elderly people and help them to live independently.

**Figure 2:**
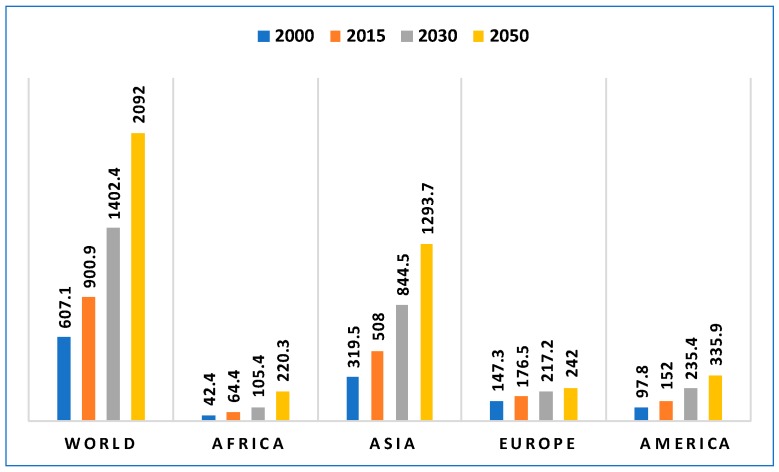
Numbers of persons (millions) aged over 60 years in the world in different years [1].

**Figure 3:**
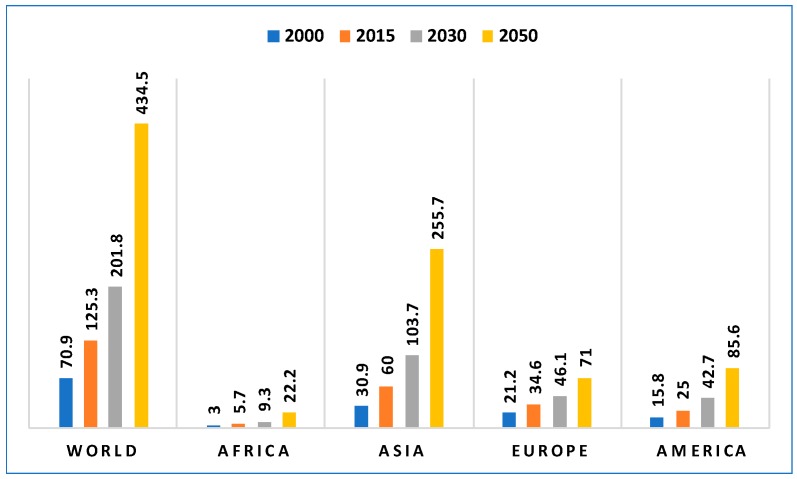
Numbers of persons (millions) aged over 80 years in the world in different years [1].

Between the years 2015 and 2030, the global population aged 60 years or over is predicted to grow from 901 million to 1.4 billion [1]. By 2050, it is predicted to have more than doubled in size relative to 2015, to nearly 2.1 billion. The number of people in the world who are aged 80 years or over is growing even faster. Projections indicate that in 2050, the number of people who are aged 80 years or over will be 434 million. This is more than *triple* the number in 2015, when there were 125 million people aged 80 years or over. Figure 2 shows the projected world population range between 2000 and 2050 for the people aged 60 years or over. Similarly, Figure 3 shows the projected world population range between the years 2000 and 2050 for people aged 80 years or over. A major challenge in handling an aging population is the effective delivery of healthcare services. Also, personal care of elderly people is a matter of great concern for their relatives, especially when they stay alone in the home and unforeseen circumstances may occur that affect their well-being. Hence, solutions are required to manage the complex care demands and to satisfy the necessities of elderly people for prolonged living in their own homes. Elderly people also have great risk of falling [2]. One of the major problems in handling this complex care is that resources are becoming scarcer day by day [3,4]. Through recent advances in sensor and communication technologies, monitoring technologies have become an important solution for achieving a robust healthcare system that can help elderly people live independently for a longer time [5,6].

Among the researchers of smart elder care systems, Celler et al. proposed one of the pioneering telemonitoring systems for remotely determining the functional health status of an elderly person [7]. The system could passively observe interactions between elderly people and their living environment over a long time. The elderly behavior monitoring system used magnetic switches to record movement in rooms, infrared sensors to detect activities, and sound sensors to determine the types of activities. Thus, it was possible for the system to respond to any activity that was outside normal activity patterns. Other technologies emerged in the following decades that focused on monitoring elderly behaviors such as daily activities and fall detection. However, an overview of non-wearable ambient sensor-based systems would be valuable for analyzing the increasingly complicated care demands of elderly people.

### 1.1. Surveys on Ambient Assisted Living

Several surveys have been conducted on sensors for ambient assisted living systems [8,9,10,11,12,13,14,15,16]. A recent survey by Al-Shaqi et al. aimed at providing a thorough review of the ambient sensor systems that are utilized in many assisted living environments [8]. The authors found that all the frameworks focused on activity monitoring for assessing immediate risks rather than identifying long-term risks in elderly care. In another survey [9], the authors proposed a classification of the activities of elderly people in smart home scenarios. In the survey, they also classified sensors that are ideal for the detection of activities.

Another survey, which was compiled by Alam et al. [10], provided a review of some smart home projects that are related to three desired services: comfort, healthcare, and security. The review also described several important components of the systems: sensors, multimedia systems, communication protocols, and algorithms. Rashidi and Mihailidis [11] also surveyed ambient assisted living technologies for elderly care. They summarized tools and technologies of smart homes, assistive robotics, and e-textile sensors. In the survey, the authors also tried to explore healthcare applications that focus on algorithms for modeling elderly behaviors in smart homes. Salih et al. [12] presented an ambient-intelligence-assisted healthcare monitoring review, in which they mostly described works that are based on wireless sensor networks technologies with applications. Furthermore, they discussed several data mining techniques for ambient sensor monitoring of patients with chronic diseases and elderly people. Peetoom et al. [13] analyzed current studies that are related to daily activity and significant event monitoring of the elderly. Their identified five main sensors types: motion, body-worn, pressure, camera, and sound sensors. Additionally, they discussed the outcomes of adopting these sensor-based technologies for prolonging independent living of elderly people. Khusainov et al. [14] proposed a survey on real-time human wellness annotation. They also analyzed different algorithmic techniques on the data of different sensors to find an effective way of addressing the demands of assisted living. The survey was focused on multiple tasks, such as sensor types, frameworks, data collection, processing, and analysis. Avci et al. [15] surveyed inertial sensor-based activity recognition methods for healthcare and well-being. Although the work is nicely arranged in line with main techniques that are found in behavior monitoring systems, it only focused on inertial sensors. Bulling et al. [16] gave a detailed outline of human activity recognition but, similarly, focused on inertial sensors. The results of all these studies indicate very positive effects of health or behavior monitoring technologies on both residents and caregivers. Furthermore, among the research surveys that are related to different aspects, some fundamental issues (e.g., proper sensor selection) regarding the necessity and adaptability of elderly people have not been reported. Table 1 lists various sensors that are used in ambient assisted living and their types, characteristics, and approximate costs. The data in the table are as reported in [8].

According to Table 1, the wearable sensors are difficult to install on the body and require professional adjustments [17]. However, wearable sensor-based systems basically include various types of on-body sensors that can measure important parameters such as acceleration, velocity, magnetic forces, heart rate, body temperature, oxygen saturation, respiration rate, electrocardiogram, etc. The obtained signals can be communicated via a wired or wireless system to a central node for further processing. A wearable sensor-based healthcare system may consist of an extensive variety of components including sensors, wearable materials, actuators, wireless communication module (s), processing unit, user interface, and advanced algorithms for data processing and decision making. On the other hand, the ambient sensors are embedded into daily environments, which is in contrast to wearable body sensors. Ambient sensors usually collect various type of data to model the events or activities of the smart home users and to anticipate their necessities to maximize their quality of life [18].

### 1.2. Ambient Assisted Living Projects

During the last few decades, many researchers have tried to carry out smart home projects [19,20,21,22,23,24,25,26]. For instance, GatorTech [19] is an earlier smart home project that was developed at the University of Florida. The project adopted various ambient sensors to provide several services to the users, such as voice and behavior recognition. The CASAS project [20] was a smart home project that was carried out at Washington State University in 2007. It was a multi-disciplinary project for developing a smart home using different types of sensors and actuators. The researchers in the project adopted machine learning tools for user behavior analysis. They focused on creating a lightweight design that could be easily set up without further customization. SWEET-HOME was a French project on developing a smart home system that was mostly based on audio technology [21]. The project aimed at three key goals, which included the development of an audio-based interactive technology that gives users complete control over the home environment. The Markov Logic Network has been a prominent approach in research on smart homes for context-aware decision processes for coping with uncertain events that are predicted from sensor data [22]. In [24], several ambient sensors such as cameras and microphones were combined to recognize elderly activities (e.g., lying, sitting, walking, standing, cycling, running, and ascending and descending stairs). In [25], the authors suggested a smart assistive living environment for facilitating the prolonged stay of elderly people at home. Sensors were installed in the smart home to provide continuous data to a server. Extracting and analyzing the data using machine learning tools helped execute the diagnosis and decision-making process for caregivers and clinical experts. However, the smart home projects provide many datasets [26]. Some of them are still publicly available for smart home researchers.

**Figure 4:**
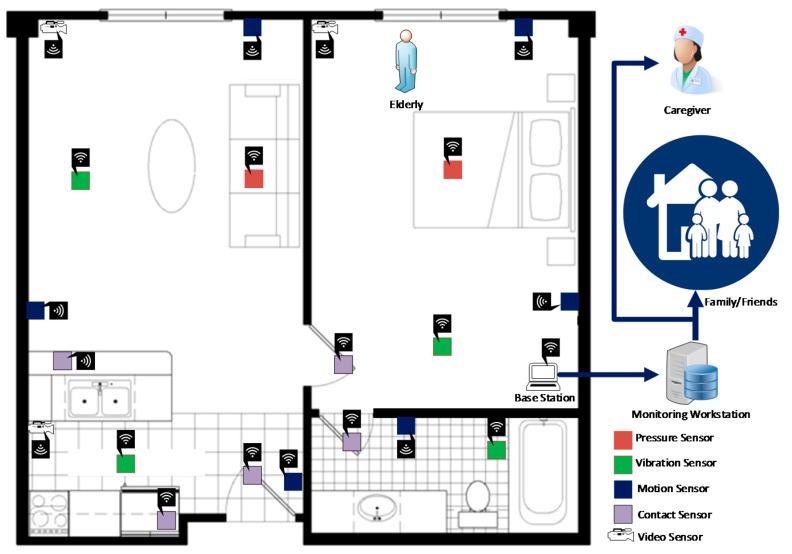
Sample schematic setup of a smart apartment for elderly care based on different ambient sensors.

**Figure 5:**
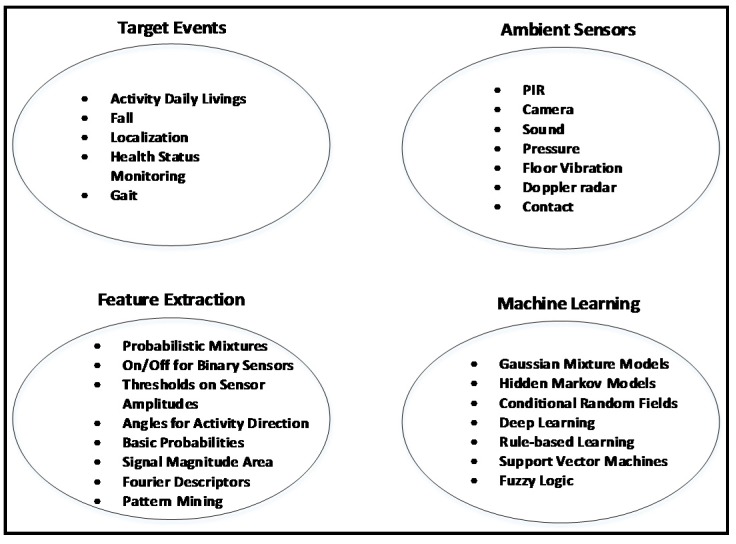
Different domains of ambient sensor-based elder care systems.

The ambient sensors that are used for elderly care can be placed in different locations in a smart home to monitor human behavior or health status. Figure 4 shows a sample schematic setup of a smart apartment for behavior monitoring of an elderly person based on different ambient sensors. Some frequently used sample sensors are shown in different places in the apartment. Other sensors can be installed, such as environmental sensors, for measuring temperature, humidity, etc. Figure 5 shows the major domains for ambient sensor-based elderly monitoring: target events, sensors, features, and machine learning.

### 1.3. Privacy and Sensitive Data Protection

Sensor-driven smart elderly health care is based on collaboration between humans and technology, where machines provide substantial support using decision support systems [27,28]. The rights and legitimate concerns of the elderly must be balanced with the requirements of efficiently functioning healthcare systems. Thus, it is very important to determine the legal obligations that may arise from privacy and personal data collection issues. An elderly healthcare project mainly involves the collection, storage, and transmission of health data.

The right to privacy is strictly connected to personhood and personal rights. Every person has the right to decide what and when monitoring data can be shared with other persons [29]. This right is connected to personal freedom and the building of identity. Currently, this right is at risk due to massive technological advancements. The technological options for monitoring have expanded enormously, especially technologies for constant monitoring of our activities, i.e., what we do and where we go. The connections between objects and persons allow for the constant monitoring of people. Besides, the data collection of sensors for monitoring physiological parameters (e.g., blood pressure, heartbeat, body temperature), behavior, and emotions generates confusion as to whether people are still able to live autonomous and free lives [30]. Thus, privacy protection is essential.

Personal data are data that relate to an individual person, who is either identified or identifiable. The primary aims are the protection of the data holder and the provision of available services to the data holder. Health-related data are data that concern all aspects of health, including physical and psychological data. Health care services require personal and sensitive data to be stored within secure information systems. Then, the data can be made available to medical professionals, users, or the user’s relatives. It is crucial to ensure that the rights of the user are protected. These technologies expose the user to serious threats, which require privacy and data protection. However, these technologies play an important role in solving the problems that they create, while enhancing technological practices with the legal necessities of privacy and personal data protection [31].

### 1.4. Article Searching Method

The search of the related research works was done in PubMed, IEEE Xplore, ScienceDirect, Web of Science, and Google Scholar. During the search, some necessary key terms were used such as “ambient sensors”, “elderly”, “aged”, “daily activities”, “environmental monitoring”, “independent living”, “smart home”, “ambient assisted living technology”, “behavior monitoring”, “activity recognition”, and “in-home monitoring” with both “AND” and “OR” connectives. The search was further extended by combining the individual sensors with the key terms. The articles that considered only wearable sensors for assisted living were ignored. However, some works were considered where wearable sensors were combined with ambient sensors.

### 1.5. Contribution and Organization of the Paper

Sensor-based surveys have mostly focused on wearable sensors or have sometimes combined them with ambient sensors to facilitate independent living of the elderly. The data collection process using wearable sensors is usually easier than that using ambient sensors. However, restrictions regarding wearing the sensors on body could discourage the elderly people from adopting them. Furthermore, there is a high possibility that some wearable sensors can generate an uncomfortable feeling during long-term skin attachment (e.g., electrodes on the skin). Hence, wearable sensor-based technologies that are used to help elderly people live independently may face a high risk of rejection, especially at home. In contrast, external or ambient sensors should be highly accepted by the elderly. However, it is important to verify that the sensors collect accurate data from a distance. Moreover, wearable sensors may require professional adjustments on the body to collect accurate data, which indicates that a complex process may be necessary for installing the sensors. Hence, considering the drawbacks of wearable sensors, reliable ambient sensors are expected to be an appropriate choice for helping elderly people live independent lives. The main contribution of this survey is the identification of research works in which mostly ambient sensors are used to obtain data from users, especially elderly users. In addition to providing references to ambient-sensor-related works, we include a summary of each work, as reported in Table 2, Table 3, Table 4, Table 5, Table 6, Table 7, Table 8, Table 9 and Table 10. The works are organized in the tables in alphabetical order of the last name of the first author. Therefore, this survey should be helpful for researchers who are working on ambient assisted living technology development to help elderly people live independent lives.

The rest of the paper is organized as follows: Section 2 includes basic descriptions of different ambient sensors and the research works that are based on them. Section 3 discusses different challenges for ambient-sensor-based projects. Finally, Section 4 presents the conclusions of the survey.

## 2. Ambient Sensors in Elderly Care

The concept of sensing arises in the smart home, in which various types of sensors/devices are integrated into everyday objects. Infrastructure in the smart home is connected by network technologies for gathering contextual information such as vital signs and behavioral information of the elderly via the sensors. The most common approaches for elderly monitoring in smart homes are based on machine vision. However, other sensors (e.g., motion, radar, object pressure, and floor vibration sensors) are also used for elderly health and behavior monitoring. In this section, we will summarize the works that apply these sensors to monitor elderly behavior or health status in recent research.

### 2.1. Passive Infrared (PIR) Motion Sensors

Many research works have applied passive infrared (PIR) motion sensors to detect the movements of individuals. PIR motion sensors are installed on walls or ceilings of the homes of elderly people to continuously collect motion data that are related to predefined activities in the scope of the sensors. PIR motion sensors are usually heat-sensitive. The sensors detect the presence of users in rooms by utilizing the changes in temperature. PIR motion sensors are used in different places to detect different types of events, such as stove use, room temperature, use of water, and opening of cabinets. Motion data are collected and transmitted to the caregivers of a user through a base station. Then, the collected data are interpreted for analysis of trends to detect changes in daily activities. They can also be analyzed to identify potential changes in health status. Thus, PIR sensors can be used to recognize patterns in daily activities and generate alerts if deviations occur. The sensors can be adopted for various applications in smart homes, such as detecting the degree of activity and detecting falls or other significant events. In most cases, monitoring technologies are combined for more than one aim, such as detecting daily activities along with significant events. Besides, PIR motion sensors can also be applied to analyze gait velocities, user location, time out of the home, sleeping patterns, and activities at night. Table 2 lists research works that were conducted based on PIR sensors [32,33,34,35,36,37,38,39,40,41,42,43,44,45,46,47,48,49,50,51,52,53,54,55].

### 2.2. Video Sensors

The most commonly used ambient sensors for eldercare are video sensors. Many research works have been carried out in ambient assistive living using video cameras for various applications, such as locating residents and recognizing their activities in their homes. Cameras are installed on the walls or ceilings to detect activity through background subtraction, body shape extraction, feature analysis, and machine learning. Among many applications, video monitoring technology has mostly been used to detect activities of daily living and falls or other significant events. Table 3 lists several research works that were conducted based on video cameras [56,57,58,59,60,61,62,63,64,65,66,67,68,69,70,71,72,73,74,75,76,77,78,79,80,81,82,83,84,85,86,87,88,89,90,91,92,93,94,95,96,97,98].

### 2.3. Pressure Sensors

Pressure sensors are applied to detect the presence of residents on chairs or in bed. They can be used to detect sit-to-stand transfers and stand-to-sit transfers. Three articles are reported in this work that applied pressure sensors in smart homes [99,100,101]. Given that all three articles focused on detecting transitions from sit-to-stand and from stand-to-sit, determining the transfer duration was the main outcome. Determining the maximum force on grab bars was also discussed. Table 4 lists some works that were conducted based on pressure sensors [99,100,101].

### 2.4. Sound Sensors

For sound recognition, sensors such as microphones are utilized to detect different events such as daily activities, e.g., the sound that is generated while handling dishes or during the falling of an object or person. In the articles that are listed in Table 5, the detection of activities of daily living, along with significant events such as falls, was the main target of the monitoring [102,103,104,105,106,107,108,109].

### 2.5. Floor Sensors

Sensing of floors plays an important role in the development of sensing environments with low invasiveness. Floor sensors can make the sensing layer invisible to the user, as the floor appears to be a traditional floor. They can be applied in various practical areas, including private and public environments. For instance, smart buildings can use floor sensors to detect the presence of people to automatically control the switches of the lighting and heating systems. In smart eldercare systems, floor sensors can be used to detect emergency situations such as falls. They can also be adopted for counting people and monitoring crowd movements during public events. Articles that explain various applications that use only floor sensors are listed in Table 6 [110,111,112].

### 2.6. Radar Sensors

Among different ambient sensors, the Doppler radar is attractive because it can detect and measure any movement in the presence of stationary clutter in the background. It achieves better perception of elderly people compared to vision-based sensors since it can penetrate strong obstacles such as furniture items and walls. Furthermore, it does not raise privacy issues for in-home monitoring and avoids the inconvenience of wearable devices [96]. In addition, Doppler radar can be used for the detection of human cardiopulmonary motion, which could provide a promising approach to overcoming the problems of false triggers. Articles that analyzed various applications of radar sensors are reported in Table 7 [113,114,115,116,117].

### 2.7. Combined Ambient Sensors

Some works combined more than one monitoring technology, such as accelerometers combined with video cameras and PIR sensors. Combinations of the multiple types of sensor technologies were very frequent and diverse in nature. The most popular combination was PIR motion sensors and video cameras. The next most frequent was a combination of pressure and PIR sensors. Using different types of ambient sensors together, improvements in quality of life were achieved within different target groups, such as residents and caregivers. The use of multicomponent ambient sensor technologies increased the sense of safety, which helped to postpone the institutionalization. Although an increase in quality of life was observed for the residents and caregivers, the growth was not substantial. However, a significant growth was noticed in the hours of informal care. Table 8 lists research works that were conducted by combining different ambient sensors [118,119,120,121,122,123,124,125,126,127,128,129,130,131,132,133].

### 2.8. Combined Ambient and Wearable Sensors

There have been many research works on elderly healthcare that are based on wearable sensors, as wearable sensors can provide more accurate information on elderly health status (e.g., heartbeat, respiration, muscle movements, and blood flow). For prolonged independent living, elderly people may not be inclined to use body-worn sensors. Hence, we focus on mostly ambient-sensor-based works in this survey. However, some research works that utilized both wearable and ambient sensors have been reported here, as shown in Table 9 [134,135,136,137,138,139,140,141,142,143,144].

### 2.9. Ambient Sensors in Mobile Robotic Systems

**Figure 6:**
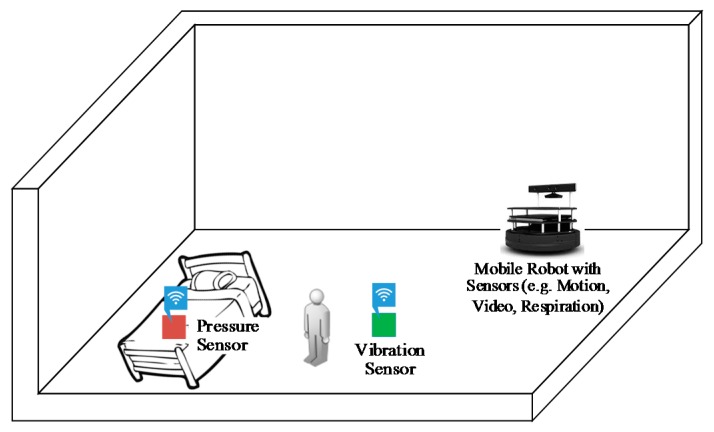
Sample schematic setup for elderly care based on different ambient sensors and a mobile robot.

Ever since the first robot was created, researchers have been trying to integrate robots into our daily lives. Domestic assistance has been a driving goal in the mobile robotics area, where robots are expected to assist in daily environments. Mobile robots can be very useful for helping elderly people live independent lives. For instance, Figure 6 shows a sample schematic setup of a smart room for behavior monitoring of an elderly person based on different ambient sensors and a mobile robot. Many mobile robots have been developed over decades by academics and research groups. The results and insights that are obtained through the conducted experiments will undoubtedly shape the care robots of tomorrow. Among all the mobile robots for elder care, several notable robots are briefly described in Table 10 [145,146,147,148,149,150,151,152,153,154,155].

## 3. Future Direction and Vision

It has been observed during the review that none of the reported works provide solutions to all the areas of ambient assisted living systems that are discussed. In many works, it is assumed that the approach is designed based on the belief that the inhabitants’ behavior is consistent every day, with the possibility of following a broad pattern. Most of the behavior models are produced based on deterministic models. The entertainment needs of elderly people have been mostly ignored. Entertainment in their daily lives should boost their lifestyles and help them enjoy their lives more. Multimedia-enabled entertainment techniques can contribute to effective treatment policy for elderly persons with memory problems. However, more rigorous study is necessary to obtain a scientific conclusion with proof. Considering the perspectives of elderly persons and caregivers, studies of this kind can help identify requirements for elderly entertainment support systems, which is challenging.

Among the many challenges that are encountered in implementing elderly assistance technology in the home, one key challenge is related to the continuous observation of the vital signs and behaviors of elderly subjects through non-wearable ambient sensors. The challenge is related to important factors such as durability, acceptability, communication, and power requirements of the sensors that are installed in the smart homes. For instance, such devices should not only provide vital signs measurements, but also deliver an assessment of the subject’s condition that is clinically correct. The sensors also should be versatile in design, with minimum weight and skin effects. The adaptability of different system components, such as communication protocols and subject interaction methods, can also be considered an important factor for ambient assistive living systems. Analyzing such factors should help system designers provide the necessary sensors and devices based on the requirements of elderly persons.

Ambient sensors such as a magnetic switch, temperature, photo, pressure, water, infrared motion, force, smoke, Doppler radar, wireless surveillance camera, and sound sensors are basically installed in several places in a smart home to acquire user data and send it to the base machine using wireless communication for further processing. The machine then typically processes the data via feature extraction and machine learning to decide the status of the user. The camera-based research works that are reported here mostly discussed the features and machine learning techniques, rather than the connectivity of the cameras. However, the depth cameras are usually connected to a machine via a wired connection to simultaneously capture the color and depth information of the user’s environment.

Beyond the works that have been reported in this review, other recently-developed sensors can be investigated for more efficient eldercare. One of the prominent candidates in this regard is the Xethru^®^ ultra-wideband radar sensors (Novelda, Oslo, Norway) for the real-time occupancy, sleep, and respiration monitoring of the elderly [165]. The radar in Xethru^®^ is a complete complementary metal–oxide–semiconductor (CMOS) radar system integrated on a single chip that is used to implement a high-precision electromagnetic sensor. The sensor can be very useful for various practical applications such as vital sign monitoring of elderly, personal security, environmental monitoring, industrial or home automation. Besides, the non-intrusive Xethru^®^ sensors can be adopted to collect relevant data while preserving the privacy of the user. Hence, they can contribute to improve the quality of life, personal comfort, and safety. The technology in these sensors combine the traditional sensor functions into one such as detecting the occupancy, calculating distance, proximity, gestures. Besides, the electromagnetic radar signals can be further explored to go through different materials such as wall to perceive the presence in next room. Hence, Xethru^®^ sensors seem to be very prominent future ambient sensors to monitor users including elderly in smart homes. Besides ultra-wideband Xethru^®^ radar sensors, WiFi signal-based ambient devices can also be explored for elderly healthcare. For instance, in [166], the authors proposed a WiFi signal-based respiration monitoring ambient device where the device was installed in a bedroom to monitor a person’s sleep.

Big data seems to transform smart homes and ambient assisted living services, especially from managerial and economic aspects. Smart healthcare projects often try to utilize consumer targeted technology. The technology includes a set of sensors and devices for monitoring the users and prediction of problems related to emergency cases. At the same time, the volumes of sensor data rapidly grow which makes the data more complicated and difficult to manage. Big data from ambient sensors rise to four key challenges [167]. Firstly, the accuracy of technologies to capture, store, distribute, and manage the information collected. Secondly, the accessibility to the large volume of data. Thirdly, managerial issues when individuals in the system change their roles. Finally, economic issues to be handled due to changes in the policies and principles of healthcare systems.

Regarding commercial implementation of sensor-based assisted living technologies, there can be many hurdles, especially for the elderly people who have dementia or Alzheimer’s disease. In addition, there is a lack of suitable outcomes to validate the installation, management, and delivery of technological solutions to meet specific needs. Insufficient experience with smart healthcare initiatives has demonstrated that pilot projects do not always lead to an extensive scale of technology applications. Thus, there are commercial concerns for providing smart home solutions, especially for people who need special assistance [168].

Elderly people are generally aware of privacy risks and possible intrusion. Acceptance of sensors such as video cameras may be challenging, as cameras can easily be perceived as intrusive by the elderly. Most researchers of elderly care in smart homes assume that users would accept the machine in the way that it is designed. With limited literature on acceptance by monitored subjects, the assumption of sensor acceptance by the elderly has not been well investigated.

Acceptability is culture-dependent and will differ from one society to another. A very important challenge for smart home system designers and developers is identifying the degree of user acceptance. In addition, there is commercial concern regarding smart home solutions for individuals with special needs.

## 4. Conclusions

This survey of ambient assisted living works has been carried out with the aim of supporting the elderly in living independent lives, mostly based on ambient sensors. It could also be helpful in supporting caregivers, friends, and family and in avoiding unexpected harm to the elderly. By far, the sensor-based surveys have majorly focused on wearable sensors alone or wearable sensors in combination with ambient sensors for the elderly. One major disadvantage of using wearable sensors is that they can generate uncomfortable feelings during extended wearing on the body, which results in a high risk of rejection by the elderly, especially at home. In contrast, ambient sensors are free from this drawback, which results in high acceptance by the elderly if they can provide reliable data. Hence, this survey has been performed by focusing on research works that are based on using ambient sensors for monitoring the health or behaviors of the users, especially the elderly. Findings from this survey also indicate that most of the frameworks on ambient assistive living primarily focus on monitoring basic daily activities and falls, while mostly overlooking the opportunities of long-term care. The potential for non-intrusive ambient sensors in elderly care is yet to be fully appreciated. Developments in low-cost embedded computing and miniaturization of electronic devices have significant potential for remarkable future advancements in the area. Future elderly care systems can also consider issues such as vital sign (e.g., heart rate and respiration) monitoring using different non-intrusive ambient sensors (e.g., ultra-wideband radar and WiFi-based sensors), providing privacy and security.

## Figures and Tables

**Table 1:** Ambient assistive living sensors, their types, characteristics, and costs, from [8].

Sensor	Type	Characteristics	Cost ($)
Magnetic switch	Ambient	The binary-status-providing sensors are easily installable. They are mainly used to detect the opening of doors, windows, etc.	5 ± 0.75
Temperature sensor	Ambient	The continuous-data-providing sensors detect the temperature of the ambient environment.	9 ± 2
Photosensor	Ambient	The sensors detect illuminance and provide continuous data.	5 ± 1.25
Pressure pad sensor	Ambient	The sensors provide continuous pressure measurement at any surface.	25 ± 5
Water flow sensor	Ambient	The sensors continuously measure the flow of water in taps or showers.	24 ± 3
Infrared motion sensor	Ambient	The binary-status-providing sensors detect motion in the coverage area.	35 ± 2
Force sensor	Ambient	Detects movement and falls	33 ± 5
Smoke sensor	Ambient	The binary-status-providing sensors detect smoke in the environment.	18 ± 6
Biosensor	Wearable	The sensors monitor vital signs and require professional adjustment. They are difficult to install.	180.00 ± 5.00

**Table 2:** Summary of research works that use passive infrared motion sensor technology.

Research Authors (Year)	Target	Research Techniques	Results
Alwan et al. [32] (2005)	Recognition of activities of daily living	The work used the following approaches: Rule-based recognition of activities (e.g., eating and showering); Fifteen on/off switches in different places, such as the microwave oven and different doors; Binary features (on/off) were used for rule-based recognition of activities of daily living; More than five weeks of activity monitoring; Subjects were provided portable personal digital assistant (PDA) devices for recording ground truth data.	91% sensitivity; 100% specificity.
Austin et al. [33] (2011)	Gait analysis	The work used Gaussian mixture modeling on motion sensor data for three years of residence monitoring of different people.	95% accuracy.
Austin et al. [34] (2011)	Gait analysis	The authors applied Gaussian-kernel-based probability density functions for three years of monitoring of two elderly subjects.	The approach detects abrupt changes in gait function and slower variations of gait velocity over time.
Barger et al. [35] (2005)	Recognition of activities of daily living	The work described probabilistic mixture model raw motion sensor data for recognition of different activities. Subjects were monitored for 65 days. Then, results were accumulated. The project utilized of a set of low-cost motion sensors. Two types of evaluations were performed: work and off-days.	The motion sensor data were grouped into 139 clusters. The experimental results showed that there were some frequent clusters that occurred consistently over time with low classification uncertainty.
Celler [36] (1995)	Recognition of activities of daily living	It was a pilot project with five months of monitoring the functional health status of the elderly at home. Parameters that are sensitive to changes in health were continuously recorded.	The project explained the technical functionality for monitoring the functional health status of the elderly in the smart home.
Cook & Schmitter-Edgecombe [37] (2009)	Recognition of activities of daily living	The work adopted Markov models for modeling daily activities.	98% accuracy.
Dalal et al. [38] (2005)	Recognition of activities of daily living	The work adopted rule-based recognition based on correlation algorithms. Each elderly person was monitored for 37 days.	91% sensitivity; 100% specificity.
Demongeot et al. [39] (2002)	Recognition of activities of daily living	The authors applied mostly threshold features for rule-based recognition.	Only analytical studies were performed, rather than reporting accuracies of proposed approaches.
Fernandez-Llatas et al. [40] (2010)	Recognition of activities of daily living	Simple rules were applied to an ongoing project to focus on various daily activities.	The work was only an analysis of an ongoing project, which was carried out to test different approaches without reporting any specific results.
Franco et al. [41] (2010)	Recognition of activities of daily living	The work used circular Hamming distance based on temporal shift, which was applied to monitor elderly persons for 49 days.	Different days were considered to explain the functionality.
Glascock & Kutzik [42] (2006)	Recognition of activities of daily living	The work applied Gaussian mixtures to model human activities. The study was performed on two field sites, where elderly monitoring was carried out for a half a year and a full year.	98% reliability.
Glascock & Kutzik [43] (2000)	Recognition of activities of daily living	Multiple activities were annotated based on specific software to monitor behavior. Elderly monitoring was performed for 12 days.	The functionality of the behavior monitoring system was elaborated for different days. It can be used in eldercare centers to obtain temporal information based on behavioral variations.
Hagler et al. [44] (2010)	Gait recognition	A simulation study was performed on gait analysis in a predefined laboratory setting.	98.9% accuracy.
Hayes et al. [45] (2004)	Recognition of activities of daily living	A Gaussian-kernel-based approach was described that was based on probability density functions for describing walking in-home. Eight weeks of monitoring of walking was carried out.	98.1% accuracy.
Kaye et al. [48] (2010)	Recognition of activities of daily living and gait	For an average of 33 months, different types of sensors were installed in the homes of 265 elderly people. Different metrics were assessed, such as total daily activity, time out of the home, and walking speed. Participants were also assessed yearly with questionnaires, physical examinations, and neuropsychological tests.	Elderly people left their homes twice a day on average for approximately 208 min per day. Average in-home walking speed was 61.0 cm/s. They spent 43% of days on the computer for an average of 76 min per day.
Lee et al. [49] (2007)	Recognition of activities of daily living	A behavioral monitoring system was developed for elderly people who are living alone. The PIR-sensor-based in-house sensing system could detect the motion of an elder and send the data to a database. In addition, a web-based monitoring system was developed for remote monitoring of the elderly by caregivers. The system was installed in nine elderly homes for three months.	86.6% accuracy.
Noury & Haddidi [50] (2012)	Recognition of activities of daily living	A simulator was proposed that focuses on human activities based on presence sensors in the smart home for elderly healthcare. Previously recorded real activity data were used to build a mathematical model that was based on HMMs for producing simulated data series for various scenarios. In addition, similarity measurements were obtained between real and simulated data.	99.91% accuracy.
Shin et al. [51] (2011)	Recognition of activities of daily living	Several sensors were installed in different places in a smart home to monitor abnormal activity patterns. Observations were made for 51 and 157 days.	90.5% accuracy.
Tomita et al. [52] (2007)	Recognition of activities of daily living	A case study was performed for two years of elderly monitoring in smart homes.	91% recommendation.
Virone [53] (2009)	Recognition of activities of daily living	It was a simulated case study in which a pattern recognition model for daily activity monitoring was tested. Activity deviation was also considered during activity monitoring.	98% accuracy.
Wang et al. [54] (2012)	Recognition of activities of daily living	Activity pattern deviations were considered for early detection of health changes. Dissimilarities among different activity density maps were computed to automatically determine changes in activity patterns. Elderly subjects were monitored for one, four, and three months.	Dissimilarities among activity density maps were in the range of 0.30–0.52.
Willems et al. [55] (2011)	Recognition of activities of daily living	A pilot study was performed to examine potential effects of activity monitoring on users and formal and informal caregivers. The study was performed based on the observations from two years of monitoring. Various questionnaires were used to assess quality of life and health status.	The functionality of the system was illustrated in detail. After the assessment, no significant variations were found based on the client questionnaires.

**Table 3:** Summary of research works that use video sensor technology.

Research Authors (Year)	Purpose	Characteristics	Outcomes
Abidine et al. [56] (2015)	Recognition of activities of daily living	The work proposed principal component analysis, independent component analysis, and linear discriminant analysis features with weighted support vector machines. The work also applied the features with other machine learning algorithms such as conditional random fields.	94% accuracy.
Aertssen et al. [57] (2011)	Recognition of activities of daily living	Motion information was extracted using motion history images and analyzed to detect three different actions for elderly people: walking, bending, and getting up. Shape deformations of the motion history images were investigated for different activities and used later for comparison in-room monitoring.	94% accuracy.
Auvinet et al. [58] (2008)	Fall detection	One of the authors of the work performed the falls on a mattress in a laboratory. The work mainly focused on post-fall phase. Twenty-two fall events were recorded for the experiments.	Analytical study of the proposed design was done rather than reporting accuracy.
Auvinet et al. [59] (2011)	Fall detection	The authors first recorded a dataset of videos from eight different cameras installed around the room where falls were simulated with the help of a neuropsychologist. For testing, some fake falls were also recorded.	100% accuracy.
Belshaw et al. [60] (2011)	Fall detection	Two in-home fall trials were done in two real living rooms. For each trial, the users performed simulated falls and real daily living behaviors for seven days. For the second trial, the users were instructed to simulate falls only and 11 simulated falls were done for seven days.	100% sensitivity; 95% specificity.
Belshaw et al. [61] (2011)	Fall detection	An annotated training set was designed with fall or no-fall. For experiments, three office rooms were set for recording training and testing videos of simulated falls over the course of three weeks.	92% sensitivity; 95% specificity.
Berlin & John [62] (2016)	Recognition of activities of daily living	Harris corner-based interest points and histogram-based features were applied with deep neural networks to recognize different human activities. The dataset consisted of six types of different activities: shake hands, hug, kick, point, punch, and push.	95% accuracy.
Brulin et al. [63] (2012)	Activity posture recognition	Fuzzy rules were applied to recognized different kind of postures: sitting, lying, squatting, and standing.	74.29% accuracy.
Chen et al. [64] (2016)	Recognition of activities of daily living	Action graph of skeleton-based features were extracted and applied with maximum likelihood estimation. Twenty different actions with 557 sequences were tried. The experiments included the cross-subject test where half of the subjects were applied for training and the rest for testing. The experiments were repeated 252 times with different folds.	96.1% accuracy.
Chia-Wen & Zhi-Hong [65] (2007)	Fall detection	The authors recorded a total of 78 videos for fall detection where 48 were used for training and 30 for testing. They focused on three feature parameters (i.e., the centroid of a silhouette, the highest vertical projection histogram, and the fall-down duration) to represent three different motion types (i.e., walk, fall, and squat).	86.7% sensitivity; 100% specificity.
Du et al. [66] (2015)	Recognition of activities of daily living	Skeleton data was extracted by sub networks and then applied with hierarchical bidirectional recurrent neural network. More than 7000 images were used to determine the postures from different activities such as undetermined, lying, squatting, sitting, and standing.	100% accuracy.
Foroughi et al. [67] (2008)	Fall and activities of daily living recognition	The authors applied best-fit approximation ellipse of silhouette, histograms, and temporal variations of head position features to represent daily activities and falls. Fifty subjects were used to record 10 activities five times each for experiments.	97% accuracy.
Huang et al. [68] (2016)	Recognition of activities of daily living	Lie group features were extracted and applied with Lie group network for different human activity recognition. The experiments included the largest 3D activity recognition dataset consisted of more than 56,000 sequences from 60 different activities performed by 40 different subjects.	89.10% accuracy.
Krekovic et al. [69] (2012)	Fall detection	The fall detection system consisted of background estimation, moving object extraction, motion feature extraction, and finally, fall detection. Dynamics of human motion and body orientation were focused. The small data set was built.	90% accuracy.
Lan et al. [70] (2015)	Recognition of activities of daily living	Dense activity trajectory was developed using histogram of oriented gradients and histogram of optical flow features to apply with support vector machines. The proposed method was validated on four different challenging datasets: Hollywood2, UCF101 and UCF50, and HMDB51.	94.4% accuracy.
Li et al. [71] (2016)	Recognition of activities of daily living	Vector of locally aggregated descriptor features were applied to analyze deep dynamics of the activities and later combined with deep convolutional neural networks. The proposed approach was tried on a public dataset of 16 different activities.	90.81% accuracy.
Li et al. [72] (2012)	Fall detection	The experimental dataset used in the work consisted two kinds of activities: falls and non-falls. The subjects were trained by nursing collaborators to act falling like an elderly. The first dataset was recorded in a laboratory where a mattress was used to fall on. The dataset consisted on 240 fall and non-fall videos (i.e., 120 for each). The second dataset was recorded in a realistic environment in four different apartments where each subject performed six falls on a mattress.	100% sensitivity; 97% specificity.
Lee & Mihailidis [73] (2005)	Fall detection	Trials for experimental analysis were done in a fake bedroom setting. The room consisted of a bed, a chair, and random bedroom furniture. The subjects were asked to complete five scenarios, which generated a total of 315 tasks consisting of 126 falls and 189 non-falls.	77% accuracy.
Lee & Chung [74] (2012)	Fall detection	Kinect depth camera with a laptop was installed to record a total 175 videos of different fall scenarios in indoor environments.	97% accuracy.
Leone et al. [75] (2011)	Fall detection	A geriatrician provided instructions for the simulation of falls which were performed using crash mats and knee or elbow protectors. A total amount of 460 videos were simulated of which 260 were falls. Several activities of daily living were stimulated other than falls to evaluate the ability of discriminating falls from activities of daily living.	97.3% sensitivity; 80% specificity.
Mirmahboub et al. [76] (2013)	Fall detection	The experimental dataset consists of 24 scenarios. In each scenario, a subject performed activities such as falling, sitting on a sofa, walking, and pushing objects. All activities were performed by one subject with different dresses.	95.2% accuracy.
Mo et al. [77] (2016)	Recognition of activities of daily living	Robust features were automatically extracted from body skeletons. The features were then applied with deep convolutional neural networks for modeling and recognition of 12 different daily activities.	81.8% accuracy.
Nyan et al. [78] (2008)	Fall detection	A total of 20 sets of data were recorded for different activities such as forward fall, backward fall, sideways fall, fall to half-left, and fall to half-right. Subjects were also asked to simulate activities of daily livings.	100% accuracy.
Peng et al. [79] (2014)	Recognition of activities of daily living	Space-time interest points, histogram of oriented gradients, and histogram of optical flow features were applied with support vector machines. The proposed approach was tried on three different realistic datasets: UCF50, UCF101, and HMDB51.	92.3% accuracy.
Peng et al. [80] (2014)	Recognition of activities of daily living	Robust dense trajectories were encoded with stacked Fisher kernels and applied with support vector machines for activity recognition. The approach was tried on three large datasets collected from different sources such as YouTube.	93.38% accuracy.
Rougier et al. [81] (2011)	Fall detection	Shape matching technique was applied was used to track a silhouette from a video sequence. Then, Gaussian mixture model was used for fall detection.	100% accuracy.
Shahroudy et al. [82] (2015)	Recognition of activities of daily living	Robust features were extracted using histogram of oriented gradients and histogram of optical flows. The features were then applied with support vector machines. The method was evaluated on three datasets: MSR-DailyActivity, MSR-Action3D, and 3D-ActionPairs dataset.	81.9% accuracy
Shi et al. [83] (2016)	Recognition of activities of daily living	Three sequential deep trajectory descriptors were tried with deep recurrent neural networks and convolutional neural networks for efficient activity recognition. The approach was tried on three datasets: KTH, HMDB51, and UCF101.	96.8% accuracy.
Shieh & Huang [84] (2012)	Fall detection	Subjects were requested to perform different events of falls and non-falls. The non-fall events include walking, running, sitting, and standing. The fall events include slipping, tripping, bending and fainting in any directions. In the experimental analysis, a total of 60 and 40 videos were used for non-fall and fall, respectively.	90% accuracy.
Simonyan & Zisserman [85] (2014)	Recognition of activities of daily living	Optical flow based temporal streams were applied with deep convolutional neural networks to model different human activities. The method was tried on two different datasets of benchmarks where it showed competitive performance with the state of the art methods.	88.0% accuracy
Uddin. [86] (2017)	Recognition of activities of daily living	Body parts in the depth images were first segmented based on random forests. Then, body skeletons were obtained from the segmented body parts. Furthermore, the robust spatiotemporal features were extracted and applied with hidden Markov models. The approach was tried on a public dataset of 12 human activities to check its robustness.	98.27% accuracy.
Uddin et al. [87] (2017)	Recognition of gaits	Spatiotemporal features were extracted using local directional edge patterns and optical flows. Then, deep convolutional neural networks were applied on them for normal and abnormal gait recognition.	98.5% accuracy.
Uddin et al. [88] (2017)	Recognition of activities of daily living	Body parts were segmented to get skeletons in the depth images based on random features and forests. Furthermore, spatiotemporal features were extracted based on the skeleton joint position and motion in consecutive frames. The body limbs were represented in spherical coordinate system to obtain person independent body features. Finally, the features were applied with deep convolutional neural networks on a public activity dataset of 12 different activities.	98.27% accuracy.
Veeriah et al. [89] (2015)	Recognition of activities of daily living	Normalized pair-wise angles, offset of joint positions, histogram of the velocity, and pairwise joint distances were applied with differential recurrent neural network. The approach was applied to recognize activities in two public datasets: MSR-Action3D and KTH.	93.96% accuracy.
Wang et al. [90] (2014)	Recognition of activities of daily living	Local occupancy patterns were applied to obtain depth maps. Fourier temporal pyramid was used for temporal representations of activities. Finally, the features were applied on support vector machines to characterize 12 different activities in a public dataset.	97.06% accuracy.
Wang et al. [91] (2016)	Recognition of activities of daily living	Weighted hierarchical depth motion maps were applied on three-channel deep convolutional neural networks. The method was applied on four different public datasets: MSRAction3D, MSRAction3DExt, UTKinect-Action, and MSRDailyActivity3D.	100% accuracy.
Wang et al. [92] (2015)	Recognition of activities of daily living	Pseudo-color images on three-channel deep convolutional neural networks were utilized to recognize activities on four public datasets (i.e., MSRAction3D, MSRAction3DExt, UTKinect-Action, and MSRDailyActivity3D) where it achieved the state-of-the-art results.	100% accuracy.
Wang et al. [93] (2015)	Recognition of activities of daily living	Skeleton-based robust features were applied with support vector machines. The approach was evaluated on two challenging datasets (i.e., HMDB51 and UCF101) where it outperformed the conventional approaches.	91.5% accuracy.
Willems et al. [94] (2009)	Fall detection	Grayscale video processing algorithm was applied to detect falls in the video. Background subtraction, shadow removal, ellipse fitting, and fall detection were done based on fall angle and aspect ratio. Finally, fall confirmation was done considering vertical projection histograms.	85% accuracy.
Yang et al. [95] (2017)	Recognition of activities of daily living	Low-level polynormal was assembled from local neighboring hypersurface normal and then aggregated by super normal vectors with linear classifier. The proposed method outperformed other traditional approaches on four public datasets: MSRActionPairs3D, MSRAction3D, MSRDailyActivity3D, and MSRGesture3D.	100% accuracy.
Yu et al. [96] (2012)	Fall detection	Simulating postures, activities, and falls in a laboratory setting.	97.08% accuracy.
Zhen et al. [97] (2016)	Recognition of activities of daily living	Space-time interest points with histogram of oriented gradient features were encoded with various encoding methods and then applied with support vector machines. The methods were tried on three public datasets: KTH, UCF-YouTube, and HMDB51.	94.1% accuracy.
Zhu et al. [98] (2016)	Recognition of activities of daily living	Co-occurrence features of skeleton joints were extracted and applied with deep recurrent neural networks with long short-term memory. The proposed method was validated on three different benchmark activity datasets: SBU kinect interaction, HDM05, and CMU.	100% accuracy.

**Table 4:** Summary of research works that use pressure sensor technology.

Research Authors (Year)	Purpose	Characteristics	Outcomes
Arcelus et al. [99] (2009A)	Sit-to-stand transfer detection	Pressure sensor arrays were installed in a bed and floor. Then, pressure information over time was analyzed. The motion of the center of pressure was observed in the wavelet domain to determine whether a transfer occurred.	Older adults generated shorter sit-to-stand durations of approximately 2.88 s.
Arcelus et al. [100] (2009B)	Sit-to-stand and stand-to-sit transfer detection.	Pressure sensors were installed in the toilet on the armrests of the commode. Clinical parameters were successfully obtained from several stand-to-sit and sit-to-stand transfers. Elderly people were included in the experiments as subjects.	Clinical parameters were successfully obtained for characterizing sit-to-stand and stand-to-sit transfer sequences. Older adults took longer and used less force in both cases.
Arcelus et al. [101] (2010)	Sit-to-stand and stand-to-sit transfer detection in bedroom and toilet.	The work focused on the analysis of sit-to-stand and stand-to-sit transfers that were performed by the occupant in the bedroom and bathroom. Pressure sensors were installed in a bed and the grab bars of a toilet commode. Then, clinical feature extraction was performed to determine a warning level.	The clinically relevant features that were obtained from both bed-exits and grab bar usage showed differences between healthy adults and those with impaired mobility. The functionality of the proposed system in keeping track of potential warning signs was demonstrated.

**Table 5:** Summary of research works that use sound sensor technology.

Research Authors (Year)	Purpose	Characteristics	Outcomes
Fleury et al. [102] (2008)	Walking, bending, and sitting recognition	The work proposed stimulating activities in a laboratory setting. The case study considered one day of monitoring.	100% accuracy.
Khan et. al [103] (2015)	Fall detection	The proposed research work developed a fall detection system based on acoustic signals collected from elderly people while performing their normal activities. The authors constructed a data description model using source separation technique, Mel-frequency cepstral coefficient, and support vector machine to detect falls. The dataset used in the work consisted of 30 fall activities and 120 non-fall activities.	100% accuracy
Li et al. [104] (2010)	Fall detection	The proposed work presented an eight-microphone circular array for person tracking and fall detection. For the sound classification, the authors applied Mel-frequency cepstral coefficients. Main design features of the array were obtained by utilizing a simulation toolbox in MATLAB.	100% accuracy
Li et al. [105] (2011)	Fall detection and localization	The authors proposed an approach for improving the accuracy of acoustic fall detection based on sliding window position and duration in data. The authors found that by positioning the window at the starting position of the signal, the highest sound source localization performance was achieved. This work applied the Hilbert transform by using a finite impulse response filter on the signals.	100% accuracy.
Popescu et al. [106] (2008)	Fall detection	Five different types of falls were targeted for experiments. A nurse was assigned to direct the subjects during the recording sessions of falls. The experimental dataset consisted of six different sessions with 23 falls in total.	100% accuracy.
Popescu & Mahnot [107] (2009)	Fall detection	The proposed work investigated a one-class classifier that required only examples from one class (i.e., fall sounds) for training. Then, fall detection was carried out based on that training.	100% accuracy.
Vacher et al. [108] (2011)	Recognition of activities of daily living	The work proposed Gaussian mixture models and support vector machines for daily activity recognition. The system also tried to recognize significant events rather than daily activities.	92% accuracy.
Zhuang et al. [109] (2009)	Fall detection	The author presented a fall detection system that used only the audio signal of the microphone. The system modeled each fall segment using a Gaussian mixture model super vector. A support vector machine was combined with the model supervisors to classify audio segments into falls.	64% accuracy.

**Table 6:** Summary of research works that use floor sensor technology.

Research Authors (Year)	Purpose	Characteristics	Outcomes
Alwan et al. [110] (2006)	Fall detection	The authors used dummies of humans to simulate original fall events. The experimental tests of falls were performed on concrete floors. A dummy was used to emulate the scenario of a person falling while attempting to get out of a chair. Another dummy was used to emulate the scenario of a person falling with upright position. The experiments were repeated three times with the same dummies.	100% accuracy.
Lombardi et al. [111] (2015)	Movement detection	A data model was proposed for storing and processing floor data. The proposed approach focused on estimating the center of floor pressure based on the widely used biomechanical concept of ground reaction force. Some practical tests on a real sensing floor prototype were attempted. The novel approach outperformed the traditional background subtraction schemas for the correct detection and tracking of people.	97% accuracy.
Serra et al. [112] (2014)	Footstep recognition	An easy-to-install and unobtrusive smart flooring system was proposed based on piezoelectric polymer floor sensors. The smart flooring system was utilized for efficient human footsteps recognition based on the Pearson product–moment correlation coefficient between the testing and reference signals for similarity calculation.	99% accuracy.

**Table 7:** Summary of research works that use radar sensor technology.

Research Authors (Year)	Purpose	Characteristics	Outcomes
Forouzanfar et al. [113] (2017)	Event recognition, such as breathing and human motion	The work proposed a methodology for classifying different events, such as breathing. Many time- and frequency-domain features were derived from radar signals. Then, linear discriminant analysis was performed to reduce the dimension of the candidate feature set. Finally, Bayesian classifiers were used to detect the target events.	Breathing: 90% accuracy. Motion: 93% accuracy.
Kim and Toomajian [114] (2016)	Gesture recognition	The work applied a deep convolutional neural network for hand gesture recognition using micro-doppler signatures. Ten different hand gestures were recognized using short-time fast Fourier transform features of the radar signals.	93.1% accuracy.
Lien et al. [115] (2016)	Gesture recognition	The work utilized a millimeter-wave radar to develop a novel, robust, high-resolution, and low-power gesture sensing technology. The overall system consisted of radar design principles, high-temporal-resolution hand tracking, a hardware abstraction layer, a radar chip, interaction models, and gesture vocabularies. The system can track gestures at 10,000 frames per second.	98% accuracy.
Rui et al. [116] (2017)	Walking speed estimation	The paper proposed an algorithm for estimating the walking speed of a human using a doppler radar system. The system was designed with the aim of passive gait assessment of elderly people. Furthermore, the work analyzed zero-crossing periods of the radar signals in the time domain to improve the dynamics of the gait signature.	97% accuracy.
Wan et al. [117] (2014)	Gesture recognition	A gesture recognition system was proposed, which was based on portable smart radar sensors with high accuracy for differentiating different types of hand and head movements. The authors adopted principle component analysis in the time and frequency domains to analyze two different sets of gestures.	100% accuracy.

**Table 8:** Summary of research works that use multiple types of ambient sensor technology.

Research Authors (Year)	Purpose	Characteristics	Outcomes	Sensors
Alwan et al. [118] (2006)	Recognition of activities of daily living	Systems for detecting activities of daily living were installed in 15 assisted living units. The reports were sent to professional caregivers of the residents. Fifteen residents and six caregivers participated in the system. It was a pilot study in which monitoring was performed for three months. Quality of life was assessed using a standard satisfaction-with-life scale instrument.	There was a high acceptance rate of the system. The approach could be used for improved healthcare planning and detection of health status changes.	PIR motion sensors, stove sensor, bed pressure sensor.
Alwan et al. [119] (2006)	Recognition of activities of daily living	Activities of daily living were monitored for 26 elderly residents and 25 caregivers over four months. A standard satisfaction-with-life scale instrument was used to assess the quality of life of the elderly people and the caregivers.	Once four months of monitoring were finished, there was no significant difference in the quality-of-life scores of the elderly users and the caregivers. The system seemed to be highly acceptable.	PIR motion sensors, stove sensor, bed pressure sensor.
Alwan et al. [120] (2007)	Recognition of activities of daily living	The purpose of the work was to assess the impact of passive health status in assisted living. Two aspects were analyzed: the cost of care and the efficiencies of caregivers. Activities of daily living systems were monitored for 21 residents for over three months.	The study demonstrated that the monitoring technologies that were used in the work significantly reduced billable interventions, hospital days, and cost of care to players. Moreover, they had a positive impact on professional caregivers’ efficiency.	PIR motion sensors, stove sensor, pressure sensors.
Ariane et al. [121] (2012)	Fall detection	The proposed fall detection system was simulated by testing on scenarios in an existing data set.	89.33% accuracy.	PIR motion sensors, pressure mats.
Bemis et al. [122] (2008)	Recognition of activities of daily living	It was a case study on two residences based on seven and four months of monitoring.	The functionality of the system in detecting activities and deviations in patterns of activities was described.	Video monitoring, PIR motion sensors.
Bemis et al. [123] (2010)	Recognition of activities of daily living	The work reported the progress in sensors, middleware, and behavior interpretation mechanisms, spanning from simple rule-based alerts to algorithms for extracting the temporal routines of the users.	The functionality of the system was demonstrated.	Video monitoring, PIR motion sensors.
Celler et al. [124] (1996)	Recognition of activities of daily living	The work presented a smart home monitoring system that was based on sequences of pressure. It mainly focused on pressure transfers in the bedroom and bathroom to check whether the motion evaluation is in the normal range or not.	The functionality of the system was demonstrated. The system showed encouraging results for precise fine-grained activity monitoring systems, especially using high-precision user localization sensors.	PIR motion sensors, sound sensors, temperature sensors, light sensors, pressure sensors.
Chung et al. [125] (2017)	Sleep stage classification	A novel approach was proposed for sleep stage classification using a doppler radar and a microphone. The classification algorithm was designed based on a standard polysomnography reference-based database and medical knowledge of doctors and sleep technologists at a hospital. The algorithm outperformed commercially available products for a specific database.	100% accuracy.	Doppler radar and microphone.
Guettari et al. [126] (2010)	Localization	This work proposed a localization system that was based on a combination of infrared sensors and sound sensors. The system mainly used the azimuth angles of the sources. This multimodal system improved the precision of localization compared to a standalone system.	54% improvement was achieved using the proposed multimodal system compared to a standalone one.	PIR motion sensors and sound sensors.
Kinney et al. [127] (2004)	Recognition of activities of daily living	It was a pilot study on 19 families for activity monitoring. Monitoring was performed for six months.	The main advantage of the system was the ease of tracking the users. The main disadvantage was the annoyance that was created by false alerts. The cost was $400 to equip the home. Ninety dollars per month was the cost of maintenance.	Video camera, PIR motion sensors.
Lotfi et al. [128] (2011)	Recognition of activities of daily living	It was a case study on two dementia patients. The first patient was monitored for 20 days. The second patient was monitored for 18 months.	The system was used to identify abnormal behavior. The system demonstrated satisfactory performance in identifying health status using different ambient sensors.	PIR motion sensors, door opening sensors, flood sensors.
Rantz et al. [129] (2008)	Fall detection	A case study was performed for retrospective analysis of fall detection data.	A change of health status was detected by the system but ignored by the nurses.	Video camera, PIR motion sensors, bed pressure sensors, door sensors.
Van Hoof et al. [130] (2011)	Recognition of activities of daily living	It was a pilot study for daily activity monitoring and fire wandering detection. The system was installed in the range of 8–23 months for analysis.	Use of the proposed system improved the sense of safety and security.	PIR motion sensors, video camera.
Zhou et al. [131] (2011)	Recognition of activities of daily living	The work tried to recognize simulated activities that were monitored in testbed for a month.	92% precision; 92% recall.	Video camera, PIR motion sensors.
Zouba et al. [132] (2009)	Recognition of activities of daily living	The authors recognized simulated activities that were monitored in a laboratory setting.	62–94% precision; 62–87% sensitivity.	Video camera, PIR motion sensors.
Zouba et al. [133] (2009)	Recognition of activities of daily living	The work was focused on monitoring simulated activities in a laboratory setting.	50–80% precision; 66–100% sensitivity.	Video camera, PIR motion sensors.

**Table 9:** Summary of research works that use ambient and wearable sensor technology.

Research Authors (Year)	Purpose	Characteristics	Outcomes	Sensors
Aghajan et al. [134] (2007)	Significant event detection	A sensor network that consisted of various types of sensors was used. Based on sensor data, event detection modalities with distributed processing were applied for smart home applications. More specifically, a distributed vision-based analysis was carried out for the detection of the occupant’s posture. Then, features from multiple cameras were combined via a rule-based approach for significant event detection.	96.7% accuracy.	Accelerometer sensors, video camera, PIR motion sensors.
Bang et al. [135] (2008)	Recognition of activities of daily living	An accelerometer and environmental-sensor-based approach was proposed. Conditional probabilities were used for recognition of daily activities that combine human motion and contacts with objects.	97% accuracy.	Accelerometer sensors, environmental sensors, PIR motion sensors.
Bianchi et al. [136] (2009)	Fall detection	The study was to evaluate barometric pressure along with accelerometer-based fall detection. Signal processing techniques (e.g., signal magnitude area) and a classification algorithm (support vector machines) were used to discriminate falls from typical daily activities.	97.5% accuracy.	Accelerometer sensors and barometric pressure sensors.
Cao et al. [137] (2009)	Recognition of activities of daily living	An event-driven context-aware computing model was proposed for recognizing daily activities.	Elderly health monitoring through the proposed system showed the effectiveness of the proposed model.	Video camera, accelerometer sensors.
Hein et al. [138] (2010)	Recognition of activities of daily living	A two-fold approach was described. First, sensors were selected based on interviews of elderly people, their relatives, and caregivers. Then, based on the outcome of the interviews, a sensor-based system was utilized to recognize different daily human activities.	Maximum 96.1% sensitivity and 90.3% specificity.	Accelerometer sensors, video camera, PIR motion sensors, door sensors.
Medjahed et al. [139] (2009)	Recognition of activities of daily living	A fuzzy-logic-based approach was proposed for robust human activity recognition on simulated data.	97% accuracy.	Sound sensors, PIR motion sensors, physiological sensors, state-change sensors.
Nyan et al. [140] (2006)	Fall detection	A fall detection approach was proposed using gyroscopes. Angles from different sides were explored for accurately modelling fall detection.	Maximum 100% sensitivity and 97.5% specificity.	Gyroscopes sensors, video camera.
Roy et al. [141] (2011)	Recognition of activities of daily living	This work proposes a framework of daily activity recognition that uses possibility theory and description logic-based semantic modeling. Different machine learning approaches (e.g., Gaussian mixture models, hidden Markov models, deep belief network) were analyzed.	95% accuracy.	Pressure sensors, accelerometer sensors, video sensors, PIR motion sensors.
Sim et al. [142] (2011)	Recognition of activities of daily living	The work applied mining of correlated patterns in activity recognition systems.	The correlated activity pattern mining approach showed 35.5% higher accuracy than typical frequent mining systems.	RFID sensors, accelerometer sensors, reed switches, PIR motion sensors, pressure sensors.
Srinivasan et al. [143] (2007)	Fall detection	The system applied triaxial accelerometer and motion detector sensor data in a two-step fall detection algorithm. First, the system tried to detect falls using the normalized energy expenditure from acceleration values. Then, falls were confirmed by considering the absence of motion. Some thresholds and logic were used to detect falls.	100% accuracy for coronal falls and 94.44% sagittal falls.	Accelerometer sensors, PIR motion sensors.
Tolkiehn et al. [144] (2011)	Fall detection	The system used a 3D accelerometer and a barometric pressure sensor for robust fall detection, along with detection of the fall direction. The basic probability-based amplitude and angular features were obtained from accelerometer sensors. Later, a pressure threshold was used.	Maximum 89.97% accuracy for fall prediction and 94.12% for fall direction.	Accelerometer sensor, barometric pressure sensor.

**Table 10:** Summary of research works that use ambient sensors in mobile robotics.

Robot	Characteristics	Ambient Sensors
AIBO [145] (2008)	It is a dog-like robot that is capable of facial expressions. The companion robot can display how it feels through six emotional states: happiness, dislike, anger, love, sadness, and surprise. It has touch sensors on the head, chin, and back. Stereo microphones allow it to hear. The camera helps it to see and balance. It also uses infrared, acceleration, and temperature sensors to adapt to its surroundings.	Touch sensors, video camera, distance sensor, microphone, temperature sensor.
AILISA [146] (2005)	It is a machine-like robot. It provides mobility aid, physiological monitoring, and fall monitoring.	Motion sensors, wireless weight scale sensors.
Cafero [147]	It is machine-like robot that provides helps with monitoring and recording vital signs, telepresence, cognitive training, entertainment and reminiscence, and scheduling activities.	Infrared sensor, camera sensor, laser range finder, sonar sensors.
Care-O-bot [148] (2009)	It is a human-like robot. It provides aid with walking with navigation, fetching objects, security, monitoring health and personal safety, cleaning tasks, heating food, telepresence, and medication reminders.	3-D time of flight cameras, stereo camera, microphone sensor on robot head, tactile sensors on robot hand
GiraffPlus [149] (2014)	It is a machine-like robot with a touchscreen interface. It allows remote people (i.e., caregivers, family, and friends) to virtually visit an elderly person’s home, move the robot about freely, and communicate with the elderly person through video conferencing technology.	Passive infrared detector, electrical usage sensor, a pressure sensor (bed/sofa/chair), accelerometer sensor, physiological sensors (e.g., body weight, blood pressure, pulse rate).
Hector [150] (2013)	It is a machine-like robot with a touchscreen interface. It provides aids for recording daily routines, controlling the environment, cognitive training, reminding to take medication, reviewing of daily agendas, detecting falls, and providing help during emergency.	Kinect depth camera, web camera, fisheye camera, microphone, IR motion sensor.
Act [151] (2008)	It is a cat-like robot. Through a camera, it can recognize objects and faces. Microphones in it can recognize speech and the direction of the sound source. It can also sense touch through its touch sensors.	Video camera, touch sensor, microphone.
NeCoRo [152] (2005)	It is a cat-like fluffy robot. The tactile sensors in its head, chin, and back can sense a stroking or patting. A microphone in its head can detect sound and the source of the sound. A camera helps it avoid obstacles. An acceleration sensor helps it recognize its position while spinning around.	Video camera, touch sensor, microphone, tactile sensor, position sensor, vision light sensor.
Paro [153] (2016)	It is a seal-like fluffy robot that is capable of facial expressions. It can provide company to elderly people who are living alone. It can perceive its environment with the help of five types of sensors: temperature, tactile, light, sound, and posture sensors. Light sensors help it to recognize light and dark. Tactile sensors help it feel when it is being stroked or beaten. Posture sensors help it sense when it is being held. Sound sensors help it perceive the directions of voices and words.	Light sensor, auditory (determination of sound source direction and speech recognition) sensor, balance sensor, tactile sensor.
Pearl [154] (2002)	It is a human-like robot with a head that is capable of facial expressions. It aids in the reminding of daily agendas, guiding around the home, reminding of appointments, telepresence, monitoring of health, and opening or closing the refrigerator.	Navigation sensors that use a laser range- finder, sonar sensors, microphones for speech recognition, stereo camera systems.
Ri-man [155] (2007)	It is a human-like machine. It aids in lifting and carrying people.	Tactile sensors
MOVAID [156] (2007)	It is machine-like robot with no head and approximately two meters tall. The robot was designed for heating/delivering food, changing bed linen, kitchen bench cleaning.	Ultrasound, force, camera, local positioning, laser, infrared, and tilt sensors.
Guido [157] (2005)	It is a machine with the height of around one meter. This robot basically aids for walking and navigation.	Force, local positioning, and laser sensor.
HOMIE [158] (2005)	This robot is a dog-like robot which was designed to give company to the elderly people. It can show some emotions and also, provide entertainment and medical attendance services.	Microphone, pressure, and motion sensors.
Wakamaru [159] (2006)	It is a human-like robot where the head of the robot can perform some facial expressions. The functions of the robot include security, managing schedules, information service, face recognition, conversation, medication reminder, and reporting unusual situations.	Camera, ultrasonic, bumper, microphone, and step detection sensors.
IRobiQ [160] (2010)	It is human-like Korean robot with a static face. The height of the robot is approximately 0.3 m. The robot helps the users with medication reminders, cognitive training, entertainment, telepresence communication, and vital signs monitoring.	Touch screen, microphone, infrared sensor, ultrasonic, and camera sensors.
Ifbot [161] (2004)	It is human-like robot with a static face and height of approximately 0.3 m. The robot was designed for entertainment purposes, cognitive training, and basic health monitoring.	Ultrasonic, infrared, camera, microphone, touch, and shock sensors.
Teddy [162] (2011)	It is a bear-like companion robot which can show different kinds of emotions via facial expressions.	Touch, microphone, and camera sensors.
Huggable [163] (2006)	It is bear-like companion robot that shows some facial expressions. The simple robot can give and receive hugs. To create a sensitive skin of the robot, more than 1000 touch sensors are underneath the skin of the robot.	Cameras, touch, force, and micophone sensors.
iCat [164] (2018)	It is cat-like companion robot with some facial expressions. The height of the robot is approximately 0.4 m.	Camera, microphone, and touch sensors.
